# Quantification of the Uncertainty in Ultrasonic Wave Speed in Concrete: Application to Temperature Monitoring with Embedded Transducers

**DOI:** 10.3390/s24175588

**Published:** 2024-08-29

**Authors:** Rouba Hariri, Jean-Francois Chaix, Parisa Shokouhi, Vincent Garnier, Cécile Saïdi-Muret, Olivier Durand, Odile Abraham

**Affiliations:** 1 Univ Gustave Eiffel, GERS-GeoEND, F-44344 Bouguenais, France; olivier.durand@univ-eiffel.fr (O.D.); odile.abraham@univ-eiffel.fr (O.A.); 2 Aix-Marseille Université, CNRS, Centrale Méditerranée, LMA UMR7031, 13625 Aix-en-Provence, France; jean-francois.chaix@univ-amu.fr (J.-F.C.); vincent.garnier@univ-amu.fr (V.G.); cecile.saidi@univ-amu.fr (C.S.-M.); 3 Engineering Science and Mechanics, The Pennsylvania State University, University Park, PA 16802, USA; pxs990@psu.edu

**Keywords:** ultrasound, embedded piezoelectric transducer, monitoring, concrete properties, uncertainty

## Abstract

This article presents an overall examination of how small temperature fluctuations affect P-wave velocity (*V_p_*) measurements and their uncertainties in concrete using embedded piezoelectric transducers. This study highlights the fabrication of custom transducers tailored for long-term concrete monitoring. Accurate and reliable estimation of ultrasonic wave velocities is challenging, since they can be impacted by multiple experimental and environmental factors. In this work, a reliable methodology incorporating correction models is introduced for the quantification of uncertainties in ultrasonic absolute and relative velocity measurements. The study identifies significant influence quantities and suggests uncertainty estimation laws, enhancing measurement accuracy. Determining the onset time of the signal is very time-consuming if the onset is picked manually. After testing various methods to pinpoint the onset time, we selected the Akaike Information Criterion (AIC) due to its ability to produce sufficiently reliable results. Then, signal correlation was used to determine the influence of temperature (20 °C to 40 °C) on *V_p_* in different concrete samples. This technique proved effective in evaluating velocity changes, revealing a persistent velocity decrease with temperature increases for various concrete compositions. The study demonstrated the capability of ultrasonic measurements to detect small variations in the state of concrete under the influence of environmental variables like temperature, underlining the importance of incorporating all influencing factors.

## 1. Introduction

Concrete is the world’s most prevalent construction material thanks to its low price, high compressive strength, and relatively good durability [1]. However, concrete structures are subject to a variety of environmental and mechanical stressors during their service life that may degrade concrete’s mechanical properties over time [2,3,4,5]. This highlights the need for cost-effective methods of monitoring concrete structures to regularly, accurately, and objectively assess the state of the material without compromising structures’ integrity. The overall goal is to enable a state-aware maintenance schedule to increase the lifespan of the monitored structures while improving the structures’ reliability and safety [6].

Time-lapse ultrasonic probing with embedded transducers is an emerging monitoring modality that provides an effective means of quantitatively tracking the evolution of concrete materials’ properties [7]. In contrast to traditional Structural Health Monitoring (SHM) of concrete structures [8,9] that yield the global state of the structure, ultrasonic testing [10,11], possibly in combination with other Non-Destructive Testing (NDT) modalities [12], is well suited local evaluation of the changes in concrete material properties that are indicators of durability, namely Young’s modulus, porosity, and water content.

Different SHM methods based on the use of PlombZirconate Titanate (PZT) transducers, like the Electromechanical Impedance Method (EMI), have proven to exhibit high efficiency in detecting early damage and monitoring concrete structures [13]. For instance, the EMI technique is particularly used in identifying micro-level damage and changes in stiffness of concrete structures by measuring the electrical impedance of the PZT transducer associated with the concrete surface [14]. Despite the advantage of monitoring with minimal processing complexity, EMI methods are usually very sensitive to surface conditions and may not offer the same depth profiling ability as ultrasonic techniques. Additionally, various PZT-based SHM methods incorporate active sensing with guided waves [15], allowing them to cover larger domains of the concrete structure. However, while these techniques have proven effective for many applications, they cannot be used for detailed and localized evaluation of concrete properties.

This work is part of the French National Research Agency’s (ANR) SCaNING project titled, “Monitoring of new and existing concrete infrastructures by embedded sensors to assess the indicators for their sustainable management” and has, as its central theme, NDT. Its original objective is to model, implement, and compare the measurements obtained by ultrasonic, capacitive, and resistivity sensors embedded in the heart of the concrete and those obtained by various NDT techniques carried out on the surface of the structure. Key advances in this work include the development of custom embedded transducers and the implementation of error correction models for the qualification and quantification of uncertainties in ultrasonic velocity measurements, which enabling highly accurate and precise monitoring of the material properties of concrete over time.

Ultrasonic wave speed and amplitude are good indicators of the mechanical properties of concrete [16]. Recent advances in embedded ultrasonic sensing of concrete [17] promise an ultrasonic SHM system amenable to long-term monitoring. Such a system is capable of characterizing and profiling concrete properties at depth without losing resolution or accuracy, thereby addressing a major shortcoming of surface-mounted ultrasonic transducers. Because of their permanent installation into the structure, the use of embedded sensors offers additional advantages such as accurate transducer positioning and consistent material coupling [18].

Embedded piezoelectric transducers have been used to successfully monitor cement and concrete hydration [18], the evolution of early-age compressive strength [16,19], and the mechanical properties of concrete [9,20,21,22], as well as concrete cracking [17,23]. These transducers can operate as both actuators and receivers and achieve a good coupling with concrete and are reasonably priced. Song G. et al. [24] introduced piezoceramic–cement-based smart aggregates as multi-functional transducers for early-age concrete strength monitoring, as well as identifying the occurrence and level of impact damage in concrete. These transducers were later adopted for an active-source ultrasonic SHM system [25,26,27]. A comparative study of data collected with different smart aggregates embedded within concrete [28] revealed significant differences in the characterization of concrete’s early-age strength development. These discrepancies were attributed to the design and packaging of the considered smart aggregates. The study suggested tailoring specific packaging of the sensors to provide particular information needed at all different concrete-setting stages. This underscores the importance of careful design and calibration of embedded transducers for concrete monitoring applications.

An equally important factor in designing a reliable and effective ultrasonic SHM system is the accuracy of target measurement at depth, considering the uncertainties associated with concrete’s inherent inhomogeneity and the changes in concrete properties with environmental influences such as temperature. Many ultrasonic monitoring systems rely on the measurement of the evolution of *V_p_* [20]. One important question is what level of accuracy in *V_p_* estimation is needed for an ultrasonic SHM system to adequately monitor concrete’s durability indicators, namely water saturation and porosity. We know that for a saturation degree equal to or higher than 50%, the higher the saturation degree, the greater the increase in wave velocity; wave velocity increases by about 8% when the saturation degree in concrete increases from 50% to 100% (full saturation) [29]. In addition, when the porosity increases by about 28%, wave velocity decreases by 31% [30].

Local volumetric porosity is another significant durability indicator; an ultrasonic monitoring system should be capable of reliably mapping the porosity profile [31]. The porosity can be measured destructively with a significant precision of 0.5 percentage points using water or mercury porosimetry [32]. To achieve a comparable level of accuracy, the estimation error in wave velocity cannot exceed 1.5%, regardless of the measurement depth [33].

This study aims to develop and evaluate an ultrasonic monitoring system with embedded ultrasonic transducers capable of sufficiently accurately characterizing porosity and water content at different depths within concrete for actuator–receiver distances of about 10 cm. We focus on small-sized transducers that enable the installation of dense embedded transducer arrays for the mapping of concrete properties with high spatial resolution.

To establish measurement accuracy and precision, we quantify the uncertainties in measuring *V_p_* using our developed embedded transducers. This is carried out using an Ishikawa diagram, also known as the 5M method [34], to define and evaluate the influence quantities related to our measurements. The most significant influence quantities are identified and used to develop correction models for the measurement.

We start by describing the fabrication and installation process of our custom transducers, which are specifically designed to be embedded in concrete. Next, we present the results of a well-controlled study examining the influence of small temperature variations on the *V_p_* measured using these transducers embedded in concrete, and we underline the importance of accurately quantifying uncertainties associated with each employed measurement method. A reliable approach featuring corrections to systematically show how temperature methodically affects ultrasonic readings in concrete is presented. Finally, we conclude with a discussion of our results, including the impact of high precision in the ultrasonic measurement technique in the evaluation of concrete’s state and the implications of our findings in advancing the state of the art of ultrasonic monitoring for concrete structures.

The key innovations of this study cover the fabrication of custom-made piezoelectric transducers particularly developed for the monitoring of concrete structures and the introduction of a reliable approach that involves quantifying uncertainties in ultrasonic wave velocity measurements. This study enriches the modern techniques in SHM using ultrasonic measurement by incorporating significant influence quantities and associated correction models, leading to improvements in the detection of small variations in concrete properties. The method presented in this study enables improved interpretation and decision making for the maintenance of concrete structures, as it ensures an accurate and reliable assessment of concrete’s state over time.

## 2. Materials and Methods

### 2.1. Fabrication of an Ultrasonic Transducer to Be Embedded in Concrete

The fabrication of the transducers involved several trials to optimize their performance, with a significant challenge arising from interference noise observed in the recorded signals during testing. Previous literature and sensor fabrication endeavors have explored various methods to address potential interference issues. This interference may result from concrete’s significant water content, potentially causing a capacitive coupling between the embedded transducers. For instance, Zongjin Li [35] explained that this noise could be reduced by applying a shielding layer on the transducers to eliminate the electromagnetic effect between the transducers and concrete. In his study, Zongjin Li used silver paint as a shielding layer to eliminate this undesirable effect. However, his research demonstrates that this solution can notably reduce the amplitude of the coupled interference but cannot fully eliminate it.

In our work, it was important to address this issue comprehensively because the observed interference could coincide with the received signal, compromising accuracy in detecting the propagation time of the P wave (*t_p_*). Indeed, for a distance of 100 mm in concrete, *t_p_* is expected to be a maximum of around 22 µs, and the variation that we expect due to changes in concrete properties is less than one microsecond. This highlights the necessity of achieving high accuracy in detecting the variation of *t_p_*.

Table 1 presents different types of piezoelectric transducers used for SHM, among which PZT is the most widely used in SHM due to its high sensitivity.

Using PZT transducers offers high sensitivity and a wide frequency range, making it suitable for the accurate monitoring of concrete, despite being subjected to significant noise and needing protection layers.

For our work, we used a type of PZT patch (PIC255) due to its suitable frequency range (50–300 kHz), which fits well with our need for optimized ultrasonic measurements in concrete. Therefore, this patch is used as the central element of our transducer, which is further strengthened with additional elements like shielding layers.

**Figure 1:**
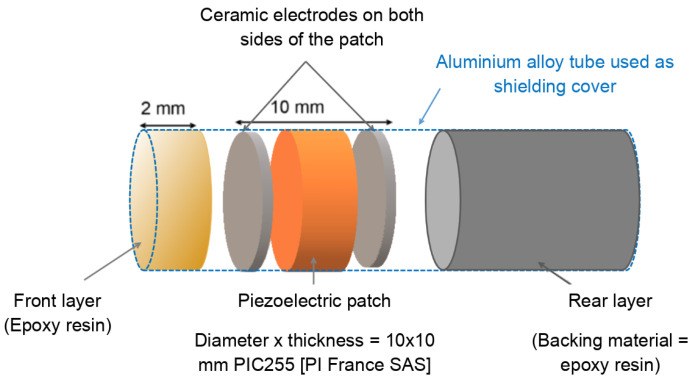
Piezoelectric transducers designed for concrete embedding. Features include a piezoelectric patch, a rear backing layer for support, a resin front layer for protection during casting, and a coaxial cable connection. The piezoelectric patch is a disk with an outside diameter of 10 mm and a thickness of 10 mm made of piezoelectric material PIC255 and a screen-printed Ag electrode [PI France SAS].

First, one concrete test cylinder was cast to verify the efficiency of the first set of embedded piezoelectric transducers, which were developed using only a piezoelectric patch coated with epoxy material to ensure the protection of the piezoelectric patch and its electrical isolation. This first set of two piezoelectric transducers (one used as a source and the second one as a receiver) was embedded in the concrete test cylinder, and the wave propagation distance between the two transducers was around 100 mm. Figure 1 shows a piezoelectric transducer highlighting its design elements.

During the test, coupled interferences were observed in the recorded signal, coinciding with the excitation time of the source signal.

**Figure 2:**
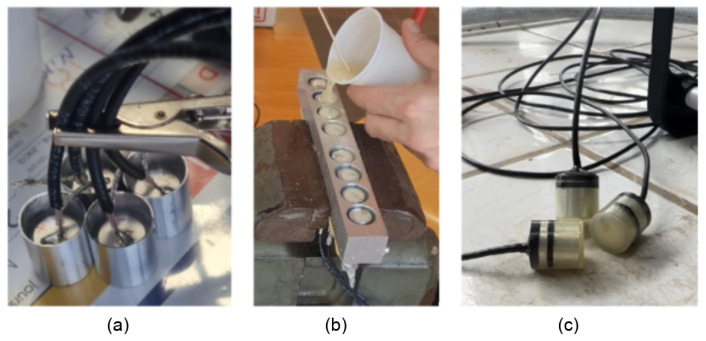
Piezoelectric transducers designed and manufactured for embedding in concrete. (**a**) The piezoelectric patch and the welded cables are set in an aluminum tube made of conductive material. (**b**) The piezoelectric patch is insulated with layers of epoxy material. (**c**) All transducers are manufactured using a digital milling machine to provide the same size for all transducers and a smooth surface to ensure good contact with concrete.

**Figure 3:**
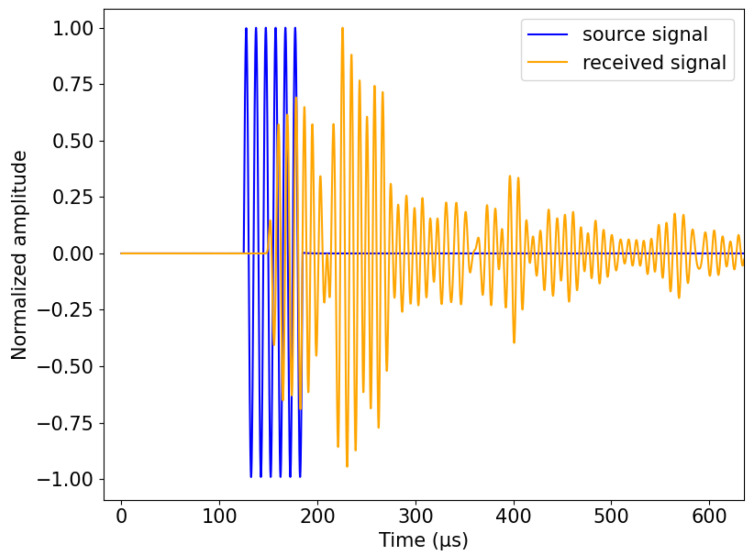
Recording of source and received signals. The blue waveform represents the source signal from the first transducer consisting of six cycles, while the orange waveform shows the signal received by the second transducer. The onset times are measured in both signals, allowing for the determination of the time of flight (TOF) by calculating the difference between the onset times. Following the P wave, subsequent wave packets containing other wave types, such as the S wave, and multiple reflections of them are observable in the received signal.

Therefore, we created a new prototype, allowing us to isolate and protect the piezoelectric patch by enclosing it in a tube made of aluminum alloy material (Figure 2a); the active part of the transducer is a piezoelectric patch made of piezoceramic material. The coaxial cable is welded to the positive and negative electrodes of the piezoelectric patch, and the electric connections are on the inside of an aluminum tube. Keeping all the cables inside the tube guarantees better coupling between the piezoelectric patch and the concrete and protects the electrical connections during installation and casting. The patch and the coaxial cable are insulated with a layer of epoxy, which is used as a backing material to dampen the resonances of the patch. As the insulation separating the power line from the instrument circuit has little effect on the capacitive coupling, the piezoelectric patch was placed with the connected cables and the backing material inside a tube made of aluminum alloy material and used as a shielding cover to ensure protection and reduce significant coupled capacitive interferences. Finally, the tube was coated with an additional layer of resin to ensure its protection, along with waterproofing and electrical insulation (Figure 2c). The coated tube is connected to the ground to allow any disruptive electrical charge to be discharged to the ground. Figure 2 illustrates the fabrication of the transducer. By implementing these details, we were able to protect the piezoelectric patch and eliminate the coupled interference caused by the electromagnetic effect. Figure 3 shows the source signal and the received signal, with a focus on the P wave’s first arrival.

In our study, we investigated various types of piezoelectric patches with different dimensions, particularly focusing on thickness variations, to determine their suitability for our application. The tested piezoelectric patches have different thicknesses (*t*) to operate in the frequency range of about one to several hundred kHz. The resonance frequencies of the transducer are governed by its thickness as follows [37]:

(1) $$t=\left(2n+1\right).\frac{Cp}{2f_{n}},$$

where *Cp* is the wave velocity in the piezoelectric material and *n = 0* is the lowest resonant frequency. The frequency range of interest is determined by the wavelength corresponding to the structure’s shortest characteristic length. In this study, the targeted applications fall within the stochastic domain, where the interaction between waves and scatterers is significant and where the wavelength (λ) is of the same order of magnitude as the size of the scatterers.

For the concrete mixtures used in this study (B30, B40, and B60), the average particle size (D) is 8 mm. Additionally, the D/λ ratio was calculated for each concrete mixture at a frequencies of 50 kHz (D/λ = 0.103 for B30, D/λ = 0.102 for B40, and D/λ = 0.126 for B60) and 300 kHz (D/λ = 0.62 for B30, D/λ = 0.61 for B40, and D/λ = 0.76 for B60). At 50 kHz, the wavelengths are notably larger than the grain size, leading to relatively low noise and, thus, minimal interference and clearer signal detection. On the other hand, at 300 kHz, the wavelengths approach the size of the grains, increasing the potential for scattering and structural noise. In summary, the D/λ ratio indicates that the structural noise is lowest at low frequencies, like 50 kHz, and it becomes higher at high frequencies, like 300 kHz. This comparison highlights the necessity of selecting the optimal frequency for embedded ultrasonic measurements in concrete, particularly when trying to balance resolution with signal precision. Additionally, the variations in D/λ ratios among the three types of concrete (B30, B40, and B60) demonstrate that each type of concrete responds differently to ultrasonic testing, with B60 possibly being the most challenging due to its high density and stiffness.

We tested various types of piezoelectric patches with different thicknesses to operate in the frequency range of about several hundred kHz ([50 kHz, 300 kHz]). The chosen patch is a piezoelectric disk with a diameter of 10 mm, a thickness of 10 mm, and a theoretical frequency of around 200 kHz.

Alongside the resonance frequency, the bandwidth of the piezoelectric path is an important parameter, as it significantly affects the resolution of the signal and, thus the accuracy in determining the arrival time of the P wave. The piezoelectric transducers developed in this study have a bandwidth of 250 kHz, spanning 50 kHz to 300 kHz. The choice of this bandwidth provides a balance between signal scattering and attenuation in concrete, since its frequency range ensures good resolution while maintaining a reasonable signal-to-noise ratio.

On the other hand, the commercial transducers used in this study have a center frequency of 58 kHz and a standard bandwidth around +/−5 kHz, presenting a limited operational range compared to the wide frequency range necessary for concrete monitoring. Although this type of transducer shows effectiveness in automotive sensors because of their high sensitivity, it may not be an optimal fit for the wider frequency range needed for accurate embedded measurements in concrete. Indeed, a limited frequency range can lead to high noise levels, restricting the transducer’s ability to filter out unwanted signals and causing it to pick up more background noise and interference from external vibrations. Furthermore, the narrow bandwidth of commercial transducers can result in limited resolution and increased noise levels when analyzing different acoustic characteristics of concrete at various depths.

When an electrical impulse is applied to the piezoelectric patch, it induces an acoustic pulse, leading to the generation of pressure waves with opposite directions on the front and back surfaces of the element. Due to its high acoustic impedance, the reverberation of acoustic waves in the patch can generate significant after-ringing, extending the duration of the acoustic pulse. Besides their mechanical purpose of ensuring the protection of the piezoelectric patch, the added layers (front and rear layers, as shown in Figure 1) are responsible for reducing the after-ringing. The first layer is used to improve the energy transmission between the active part (piezoelectric path) and the studied material (concrete). The rear layer, on the other hand, which is a thick backing layer, not only serves as a protective and mechanical support for the piezoelectric patch, as explained in Section 2.2, but also absorbs the acoustic energy to reduce post ringing.

### 2.2. Validation and Calibration of Piezoelectric Transducers

**Figure 4:**
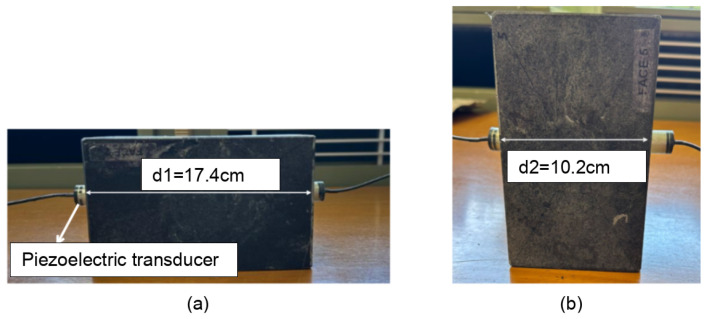
A reference material, namely marble, was used to calibrate the piezoelectric transducers. By measuring ultrasonic wave velocity at two different propagation distances d1 in (**a**) and d2 in (**b**) we can estimate the propagation time of the wave within the transducers. Note: The two depicted transducers are identical in function, although their external appearance differs slightly due to variations in the application of waterproof coating for technical purposes.

To correctly measure *t_p_* between two piezoelectric transducers, the thickness of the layers applied to the piezoelectric patch must be taken into account. Additionally, due to technical and manufacturing details, the thickness of the layers applied to each piezoelectric patch is unique for every fabricated transducer. Consequently, it is necessary to conduct a calibration procedure individually for each transducer. This ensures accurate measurement of *t_p_* between two piezoelectric transducers embedded in concrete. A marble sample (Figure 4) with ultrasonic velocity and attenuation near those of concrete was used as a reference material for the calibration process. Ultrasonic P waves were recorded at two different propagation distances (*d*1 = 17.4 cm and *d*2 = 10.2 cm), as shown in Figure 4. The reference velocity (*Vmarble*) was obtained by the measurement of propagation time at the two distances as follows:

(2) $$Vmarble=\frac{d2-d1}{t_{p2}-t_{p1}},$$

where *d*1 and *d*2 are the propagation distances measured on the marble sample, and $t_{p1}$ and $t_{p2}$ are the propagation times of the P waves measured by an oscilloscope. By comparing two different propagation distances of the same material, this measurement makes it possible to correct systematic errors like the transit time (*tc*) of the wave in the protective epoxy coating inside the ultrasonic transducer pair. *t_p_* is obtained as follows:

(3) $$t_{p}=tm-tc,$$

where *tm* is the time measured by the oscilloscope for the propagation of the P wave between the two transducers embedded in concrete and *tc* is the propagation time of the P wave inside the transducers found using the calibration procedure expressed as follows:

(4) $$tc=t_{p1}-\frac{d1}{Vmarble},$$

Using the equations presented above, the transit time of the wave inside the transducer (*tc*) is determined for each of the two developed transducers. This value (*tc*) varies among pairs, correlating with the varying thicknesses of protective layers surrounding the piezoelectric patches. However, throughout these fluctuations, a notable regularity appears with a time lag of around 0.6, μs on average. This average serves as a valuable reference point that captures the overall behavior of our transducers while highlighting the importance of considering the specific characteristics of each pair.

Marble was selected as a reference material for the calibration process due to its consistent, homogeneous nature, which is crucial for accurate calibration, as well as the similarity of its ultrasonic characteristics to those of concrete, offering a practical balance between material uniformity and the pertinence of the calibration result to the actual on-site application.

### 2.3. Concrete Specimens with Embedded Transducers

**Figure 5:**
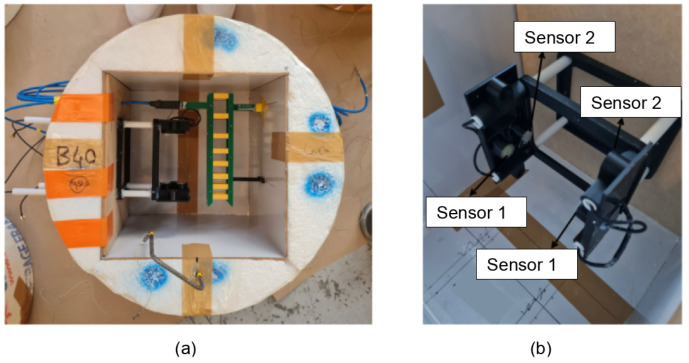
Images of the setup before concrete pouring. (**a**) Two pairs of embedded ultrasonic transducers set at the center of a concrete specimen next to an embedded electrical capacitive transducer. (**b**) Zoomed-in view of the embedded transducers (commercial ultrasonic transducers and the developed piezoelectric transducers).

Experiments were conducted on three separate cubic blocks of concrete, each representing a different mixture (B30, B40, or B60), with distinct properties (Table 2). Each individual block, corresponding to its respective mixture, is equipped with embedded transducers comprising two pairs of transducers positioned at the center of the block (Figure 5). The first pair consists of two piezoelectric transducers fabricated as shown in the previous section, while the second pair consists of transducers developed by a commercial manufacturer. The transducers (both the commercially produced transducers and our laboratory-fabricated piezoelectric transducers) were used for ultrasonic velocity measurements inside the concrete mixes. Initially, both types of transducers exhibited similar performance characteristics.

However, many weeks after casting the concrete, a significant difference in performance was observed. We noticed a reduction in signal quality recorded by commercial transducers, featuring a high level of noise preceding the signal, which affects accuracy in determining the wave travel time. In contrast, signals received by the piezoelectric transducers we fabricated were of high quality, with a superior signal-to-noise ratio. Therefore, the analysis presented in this section is based on data collected exclusively from the piezoelectric transducers developed in Section 2.1. The geometry of the blocks and the properties of the concrete mixtures are presented in Table 1. B60 exhibits superior properties in terms of density, stiffness, and compressive strength compared to B30 and B40. Therefore, B60, with the highest density, is expected to maintain the most stable *V_p_* valuesunder temperature variations, making it suitable for environments with significant thermal fluctuations. In addition to the blocks, specimens were cast for each type of concrete (B30, B40, and B60) and were subjected to mechanical testing to determine their mechanical properties, like Young’s modulus and compressive strength. The values of Young’s modulus (E) were obtained through tensile tests where each sample was subjected to increasing load. Deformation and compressive strength were calculated for each concrete sample using a compression test.

### 2.4. Conditioning Temperature and Monitoring System

**Figure 6:**
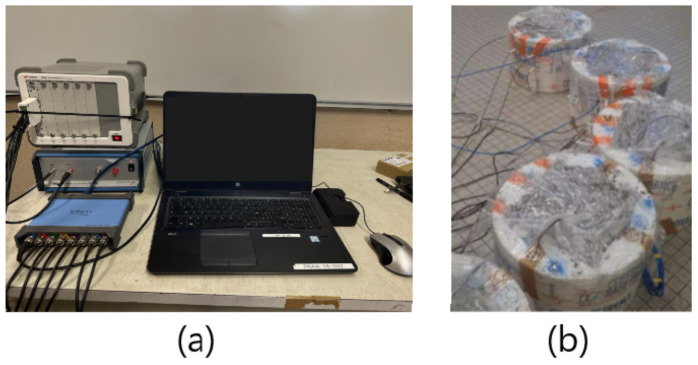
Ultrasonic monitoring system. (**a**) An eight-channel ultrasonic measurement setup for continuous real-time monitoring of concrete properties. (**b**) Presentation of coaxial cables extending from the embedded transmitter and receiver transducers to the measurement system (concrete specimens during the hardening phase). This setup is equipped with eight channels to facilitate simultaneous viewing of multiple acquisitions from the receivers.

The blocks were aged over three months before the start of the experiment. After casting, the blocks were first set in a climate chamber at 25 °C for 30 days, then kept wet for 60 days by soaking them in distilled water in order to achieve full saturation. Before being set again in the climate chamber to investigate the dependence of temperature dependence *V_p_*, the specimens were coated with aluminum foil to guarantee the stability of the water content within the blocks throughout the experiment. Temperature variation in the climate chamber was selected within a range of 20–40 °C, and the values of *V_p_* corresponding to the temperature within this range were measured continuously. The ultrasonic monitoring system (Figure 6) consists of a commercial device (keysight chassis) to synchronize multiple inputs from the source; a dual-channel, high-voltage linear amplifier; and a PC oscilloscope (PicoScope 4000A Series) with eight channels, allowing multiple acquisitions to be viewed from the receivers at the same time.

**Figure 7:**
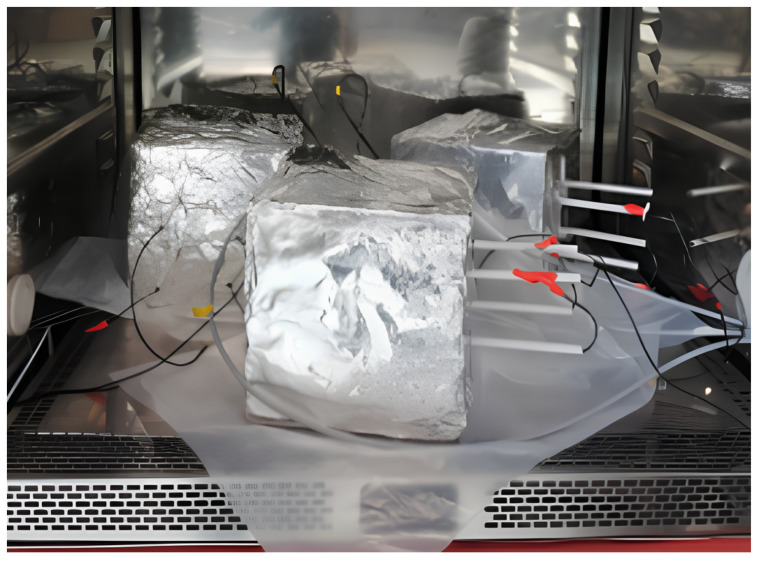
Climate chamber setup, comprising concrete blocks cast with embedded transducers protected by an aluminum foil covering to preserve moisture content.

**Figure 8:**
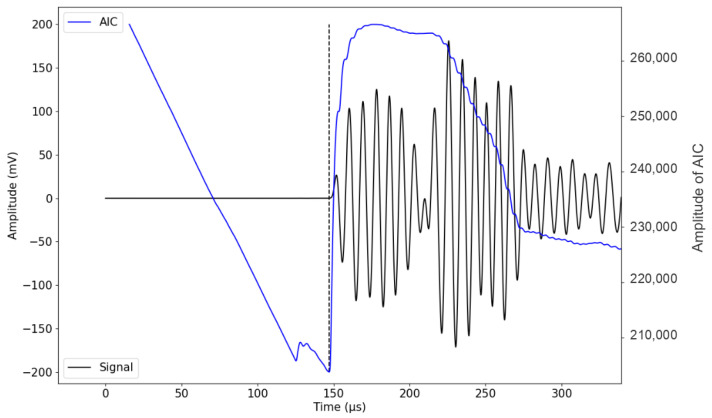
The AIC is used to determine the onset of a specific portion of the signal (solid black line). The AIC function is illustrated in blue. The intersection between the AIC’s minimum value and the signal (dashed black line) illustrates the signal’s onset time ($t_{p}$).

The measurement of *V_p_* started immediately after placing the blocks (with an initial temperature of around 25 °C) in the climate chamber (Figure 7), and results were recorded at 1 h intervals. As previously stated, our research focuses on the first part of the signal when the P wave arrives (Figure 8). *V_p_* was calculated as follows:

(5) $$V_{p}=\frac{d}{t_{p}},$$

where *d* is the distance between the transducers embedded in concrete and *t_p_* is the time of flight of the P wave (TOF). To investigate the precision of these measurements, all influence quantities are studied in Section 2.6 of this article, and the accuracy of the calculated *V_p_* is examined.

For the selection of the frequency range of the embedded source, we tested various types of piezoelectric patches with different thicknesses to operate in the frequency range of about several hundred kHz, as mentioned earlier in Section 2.1. To test the response of the chosen patch to different source frequencies, the patch was embedded in a laboratory concrete sample and used as a receiver located 100 mm away from the source. We used source frequencies in the range of 50 to 300 kHz and observed that the spectral content was significant between 100 and 150 kHz, particularly around 130 kHz. Consequently, a source frequency of 130 kHz was chosen.

### 2.5. Signal Processing for Travel Time Estimation

The source wave travels in concrete, encountering multiple obstacles (aggregates) along its path, and is turned into a complex waveform by the time it reaches the receiver (Figure 3). This complex waveform is usually found in concrete with large aggregates, where structural noise can significantly influence signal clarity.

The early part of the received waveform consists mostly of a direct P wave between the source and the receiver. Therefore, in this section, we focus on the first arrival of the signal, which carries information about *V_p_* that can be related to the mechanical characteristics of concrete.

#### 2.5.1. Determination of Onset Time

Since we are studying the influence of small temperature variations on *V_p_* in concrete, the onset time of the P-wave arrival should be determined as accurately as possible. Furthermore, the number of recorded ultrasound signals can be up to several thousand during monitoring. Therefore, it is evident that the automation of onset detection is necessary. While the use of an amplitude threshold picker is the most basic method of onset picking, it has been shown to be ineffective for low-amplitude signals and those coupled with a high noise level [38]. At low amplitudes, distinguishing between noise and true signal can become challenging, especially when the signal and the noise are in the same frequency range. Additionally, due to the significant number of received signals, applying the threshold method is time-consuming, as it requires the setting of a threshold based on noise amplitude parameters, which vary from signal to signal. Therefore, another criterion based on the shift from signal amplitude to the change in the wave energy could be used. Analyzing the signal as an autoregressive process provides an alternative technique for onset time determination. In this study, we use the AIC, which shows that a discrete time series can be divided into locally stationary segments, in which each segment is studied as an autoregressive model [39]. For a fixed order in this model, the point at which the criterion is at its minimum presents the optimal separation between the noise and the true signal (Figure 8). This criterion is expressed by Equation (6) [40,41]. To accurately determine *t_p_*, the onset times of the received signal and the source signal are measured. *t_p_* is approximately (147 −125 = 22 μs), representing a critical factor in our analysis, as it is directly related to the detection of variations in water saturation and temperature changes in concrete. For instance, temperature variations within the range of 20 to 50 °C lead to wave velocity changes of around 150 m/s equivalent to approximately 1 to 2 μs in terms of *t_p_* in concrete for a distance of 100 mm. To minimize uncertainties in time measurement, considering factors such as the heterogeneity of concrete, short propagation distance, and attenuation, the accuracy in pinpointing the arrival time of the signal is essential.

(6) AIC[k]=k.log(var(x[1,...,k]))+(N−K).log(var(x[k+1,...,N])),

*x*[*k*] is the signal at index k, N is the number of points in *x*[*k*], and var is the variance.

J. Kurz et al. [42] compared the AIC criterion to another automatic onset detection algorithm based on the Hinkley criterion, and manual time picks performed by an experienced analyst were taken as reference values. According to their results, the AIC criterion produces sufficiently reliable results for ultrasound signals, as the deviation from the manual picks varied between 2% and 4%, while the Hinkley picker showed a deviation of 17% compared to the manual picks [42]. The abovementioned study suggests that the AIC picker is a reliable tool for automatic onset detection of ultrasound signals and acoustic emissions of varying signal-to-noise ratios. Therefore, the AIC technique presents a level of accuracy that is essential for our study, where precise measurements of P-wave arrival times are necessary to investigate the influence of small temperature variations on *V_p_* in concrete.

Furthermore, the AIC technique offers high computational efficiency, which is essential when analyzing a large volume of recorded signals. Its ability to provide reliable results while requiring minimal processing time makes it well-suited for our monitoring applications, where efficiency is important. In conclusion, given the complexities of our study, including the need for accuracy in detecting onset times within noise and signal variations, the AIC technique emerges as a prudent choice for automatic onset detection in our study of ultrasonic signals in concrete. To assess the precision of our measurements, we recorded ultrasonic signals 20 times using one pair of embedded transducers in a fixed position. The AIC technique was used to accurately determine the arrival time of each signal. We estimated the precision of these measurements using a reference value as a point of comparison. The estimated calculated precision is around 1.7%, knowing that the more measurements we have, the more reliable the estimated precision will be.

(7) $$\mathrm{Estimated}\;\mathrm{precision}\;(\%)=(\frac{\mathrm{Measured}\;\mathrm{value}-\mathrm{Average}\;\mathrm{of}\;20\;\mathrm{measurements}}{\mathrm{Average}\;\mathrm{of}\;20\;\mathrm{measurements}}).100,$$

#### 2.5.2. Determining the Relative Variation of Onset Time Using Cross-Correlation

Onset time picking is subjected to some influence quantities, including the repeatability of the sample, reproducibility at different measurement points, resolution, and sampling of the oscilloscope. Additionally, the heterogeneous nature of the concrete itself has a significant impact on the arrival time of the ultrasonic signals, particularly at high frequencies. Each of these factors adds significant uncertainty to the detection of the onset time of the signals. Therefore, monitoring the changes in *V_p_* within concrete due to minor temperature fluctuations is challenging, as it requires accurate, high-precision measurements.

**Figure 9:**
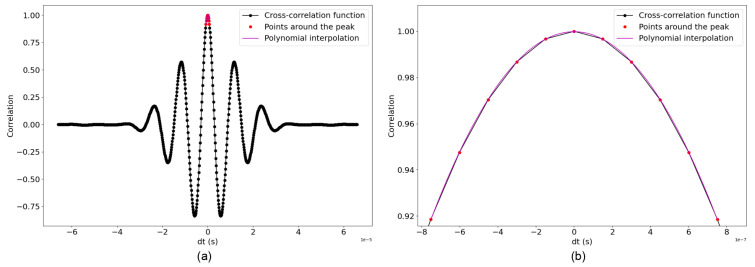
(**a**) Full cross-correlation function between two signals (at ti and To1). The points of interest around the maximum peak are marked in red, and the purple curve shows a degree 2 polynomial fitted to these points. (**b**) Zoomed-in view of the area around the peak of the cross-correlation function where the red points of interest are used to fit the 2-degree polynomial.

In this section, we present an alternative approach based on the cross-correlation technique that involves aligning the signals and analyzing the time delays between them. A reference signal corresponding to the original waveform at the beginning of the experiment was selected for comparison. This technique consists of shifting the recorded signal by a given time (τ) until an optimal correlation with the reference signal is reached. The time shift indicates the time difference between the recorded signal and the reference signal, and its value corresponds to the peak of the cross-correlation function where the two signals are most similar. By using this technique, the reference signal is correlated with the measured signals, focusing on the waveform at the start of the signal. To optimize the accuracy of this time-shift estimation, we use a polynomial interpolation around the peak of the cross-correlation function. A second-degree polynomial is used to connect the points around the peak, and this polynomial is then fitted to these points to determine the highest value of the adjusted curve, which provides a more precise estimation of the time shift dtcompared to the original sampling resolution (Figure 9). This process is essential for the evaluation of small variations in the recorded signals and, thus, potential changes in concrete properties due to environmental factors like temperature.

**Figure 10:**
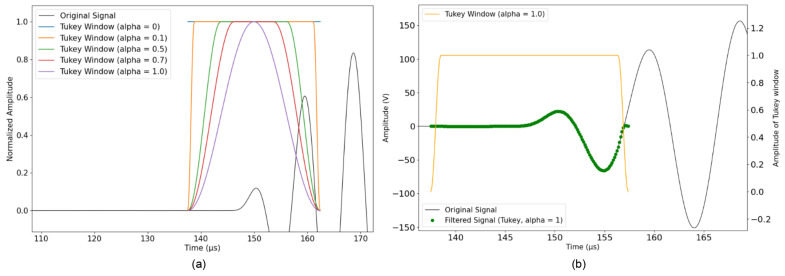
Signal recording from the transducer embedded in concrete block B30 (black). (**a**) Colored functions represent Tukey windows applied to the center of the first occurrence of the signal alternation with different alpha values. (**b**) A Tukey window with α = 0.1 is applied to the center of the first occurrence of the signal alternation (between the start point (1) and the end point (2)) with alpha = 0.1 (green).

In this study, we apply the cross-correlation method to the first part of the signal, which corresponds to the first arrival of the P wave. However, trimming a part of the signal can cause sharp edges, resulting in the introduction of discontinuities and creating edge effects. Therefore, it is necessary to use a window that reduces discontinuities at the edges of the trimmed part of the signal and improve the precision of the analysis. This processes involved using a Tukey window characterized by a tapering of the signal towards its edges to help reduce spectral absorption and improve frequency resolution. The Tukey window, also known as the tapered cosine window, can be regarded as a cosine lobe that is convolved with a rectangular window (Figure 10). Alpha (α) defines the ratio between the constant section and the cosine section. It has to be between 0 and 1. The function returns a Hann window for α equal to 1 and a rectangular window for α equal to 0. Its ‘taper length’, which determines the amount of time for which the window maintains a value of one, can be specified. The lower the taper length, the longer the Tukey window is equal to one over the measurement time.

**Figure 11:**
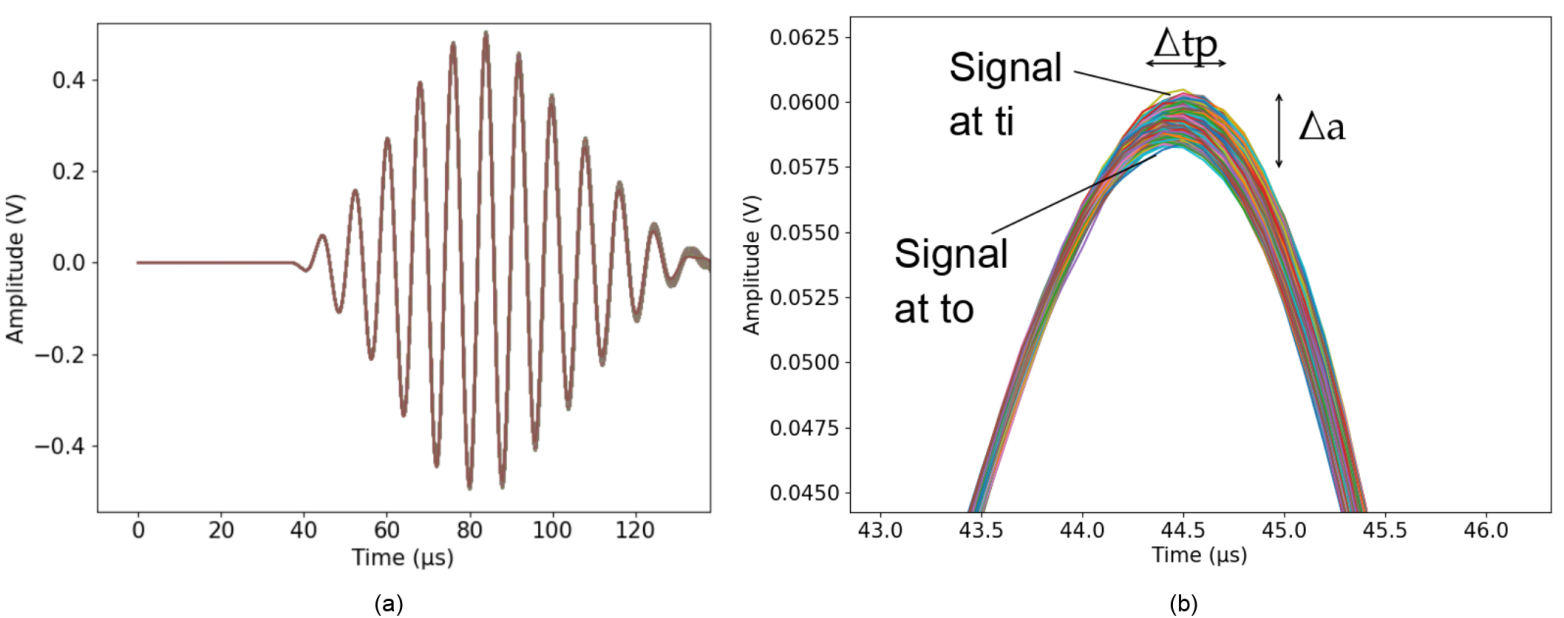
Evolution of recorded signals in time and amplitude. (**a**) Superposition of signals recorded on the piezoelectric transducer embedded in concrete block B30 during monitoring of temperature changes. The different colors represent signals captured at various time intervals or under varying conditions, highlighting the changes in signal characteristics over time. (**b**) Zoomed-in view of the start of the signal, where the variation of time ($\Delta t_{p}$) and amplitude (Δa) is detected upon the first arrival of the signal.

The advantage of a Tukey window is that the amplitude of the transient signal in the time domain is less likely to be altered than when using a Hanning or rectangular window. Because the Tukey window is close to one for a longer period of time than a Hanning window, it is better-suited to capture the amplitude of transient events. Therefore, we choose an alpha value close to 0 to keep the ’taper length’ of the window at one for the amount of time corresponding to the first arrival of the signal. Because of this choice, the constant section of the window is applied to the first arrival of the signal (Figure 10). When the temperature increases by about 10 °C, the propagation time of the wave increases by about 1%, and the amplitude increases by about 7%. On the other hand, slightly increasing the value of alpha can help to taper the signal towards its edges so that the edges of the window are not cut brutally. Therefore, we apply the Tukey window to the first arrival of the signal with an alpha value equal to 0.1. Then, a polynomial function is fitted to the interpolated data points obtained from the correlation results. The fitted polynomial is used to interpolate values through the time range of interest. Through this process, small variations in time were detected, indicating possible changes in concrete properties (Figure 11).

### 2.6. Measures and Uncertainties

**Figure 12:**
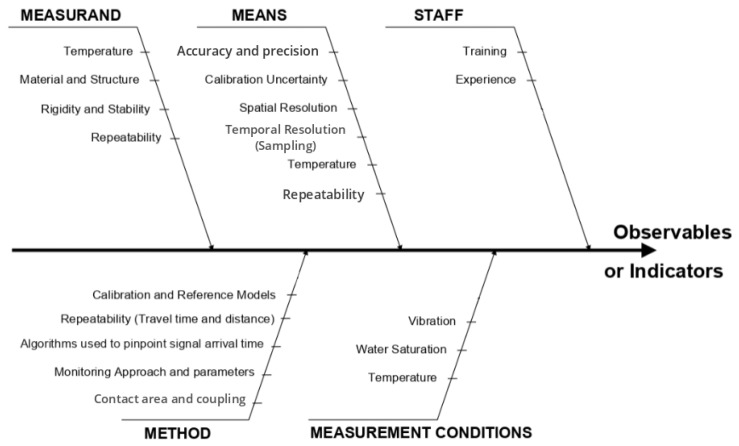
Significant influence quantities presented with and Ishikawa diagram (5M Methods) [43] for ultrasonic measurements performed using transducers embedded in concrete.

This section proposes the definition of a general methodology for the evaluation of measurement uncertainties for the following variables: *t_p_* and *V_p_*. The structural diagnosis process involves a comprehensive investigation of both the measurement method and its application to the structure, as well as a studying of the direct link between the observables and the indicators [43]. The influence quantities listed in Figure 12 are specific to ultrasonic measurements with performed transducers in embedded concrete structures.

For each observable *(y)* we assign a measured value and a corrected value, and all the errors linked to the influence quantities are included in the direct measurement as follows:

(8) $$y_{c}=y_{m}+\sum Ci,$$

where *y_c_* is the corrected result, *y_m_* is the measured value, and *Ci* is a corrective term associated with each error. Furthermore, we can quantify an expanded uncertainty *(U)* associated with the corrected value of *y_c_* (*y_c_* ± *U*) that can be derived as follows:

(9) $$U=k.u_{c}\left(y_{c}\right),$$

where *k* represents an enlargement coefficient and *u_c_(y_c_)* is defined as the composite standard uncertainty associated with the dispersion of the corrected values. This composite standard uncertainty combines all the variances associated with the influence quantities [43].

(10) $$u_{c}^{2}\left(y_{c}\right)=Var\left(y_{c}\right)=Var\left(y_{m}\right)+\sum Var(C_{i}),$$

Uncertainties in time $\left(u_{c}^{2}\left(t_{p}i\right)\right)$ and distance $\left(u_{c}^{2}\left(d_{i}\right)\right)$ are calculated using Equation (10).

#### 2.6.1. Uncertainty in Time

We start by studying uncertainties related to the onset time of P waves through concrete. It is essential to quantify the expanded uncertainty (*U_t_p__*) (Equation (9)), which is associated with the corrected value (*t_p_*), which, itself, combines all the variances of the influencing factors presented in the Ishikawa diagram (Figure 12), such as repeatability, temporal resolution, calibration, sampling, temperature, processing techniques, etc. Within this context, some quantities are essential to our study, including the repeatability of time measurements (*t_m_*), the calculation of wave propagation time within the transducers, and the temporal resolution of the signal.

(11) $$t_{p}=t_{m}+Ccalibration_{t}+Cresolution_{t},$$

(12) $$u_{c}^{2}\left(t_{p}\right)=u_{c}^{2}\left(tm\right)+u_{c}^{2}\left(Ccalibration\right)+u_{c}^{2}\left(Cresolution\right),$$

where *$u_{c}$(tm)* is the standard deviation of repeatability of time measurement (tm) in concrete. The piezoelectric transducers were first calibrated using a reference homogeneous material, as described in Section 2.2 before being embedded in concrete. Consequently, the propagation time within the transducers was calculated for each transducer pair, then used for the calculation of P-wave propagation time in concrete (Equation (3)). This calibration procedure, as detailed in Section 2.1, enabled us to account for uncertainties related to the propagation time of the wave in the layers of the transducers.

(13) $$Ccalibration_{t}=-t_{c},$$

(14) $$u_{c}^{2}\left(Ccalibration_{t}\right)=\sigma^{2}\left(t_{1}\right)+{\left(\frac{1}{C_{\mathrm{ref}}}\right)}^{2}\cdot {\left(\frac{U_{d_{1}}}{2}\right)}^{2}+{\left(\frac{d_{1}}{{\left(C_{\mathrm{ref}}\right)}^{2}}\right)}^{2}\cdot {\left(\frac{Uc_{\mathrm{ref}}}{2}\right)}^{2},$$

where $U_{d_{1}}$ and $U_{c_{ref}}$ are the expanded uncertainties of the measured distance of the marble block and the reference velocity of the ultrasonic wave propagation in marble, respectively.

$Cresolution_{t}=0$

(15) $$u_{c}^{2}\left(Cresolution_{t}\right)={\left(\frac{dt_{\mathrm{resolution}}}{2\times \sqrt{3}}\right)}^{2},$$

where ${dt}_{resolution}$ is the temporal resolution of the signal.

#### 2.6.2. Uncertainty in Distance

For distance measurements, we used a caliper with a numerical resolution of 0.01 mm, a maximum precision error of 0.01 mm, and a maximum accuracy error of 0.03 mm. The distance between the transmitter and the receiver was measured before casting. The supports, equipped with the transducers, were fixed in the blocks using PVC tubes, as shown in Figure 5 to ensure rigidity and stability of the transducer’s positions. However, this measurement could be coupled with a non-negligible uncertainty associated with some influential quantities presented in the Ishikawa diagram (using the 5M methods). Among these, certain quantities emerge as particularly pertinent to our investigation, such as measurement repeatability, resolution, precision, and accuracy errors, as well as measurement conditions like temperature. Therefore, we can calculate a corrected value of the distance and its expanded uncertainty (with *Ccalibration_d = Cresolution_d* = 0) as follows:

(16) $$d=d_{m}+Ctemperature+Ccalibration_{d}+Cresolution_{d},$$

(17) $$Ctemperature=dm.\alpha_{\mathrm{concrete}}.\left(\theta_{m}-\theta_{i}\right),$$

(18) $$\begin{array}{ccc} u_{c}^{2}\left(d\right) & = & u_{c}^{2}\left(d_{m}\right)+u_{c}^{2}\left(Ctemperature\right)+u_{c}^{2}\left(Ccalibration_{d}\right)\\ & + & u_{c}^{2}\left(Cresolution_{d}\right), \end{array}$$

(19) $$\begin{array}{ccc} u_{c}^{2}\left(d\right) & = & \sigma^{2}\left(d_{m}\right)+\left(\frac{2.d_{m}^{2}.\alpha_{\mathrm{concrete}}^{2}.d\theta^{2}}{3^{2}}\right)+\left(\frac{d_{m}^{2}.\Delta \theta^{2}.d\alpha^{2}}{3^{2}}\right)\\ & + & \left(\frac{E{\mathrm{accuracy}}^{2}}{3}\right)+(\frac{E\mathrm{precision}}{2}{)}^{2}+(\frac{Resolution}{2.\sqrt{3}}{)}^{2}, \end{array}$$

where $d_{m}$ is the measured value of distance; *Ctemperature* is a coefficient representing the effect of temperature variation on distance measurement in concrete, with $\alpha_{concrete}$ being the coefficient of thermal expansion of concrete (α = $1\times 10^{-5}$K^−1^); and dα is the uncertainty value associated with α (dα = $2\times 10^{-6}$K^−1^). $\theta_{m}$ and $\theta_{i}$ are the temperature during ultrasonic measurement *($\theta_{m}$* = 20 °C, 30 °C, and 40 °C) and the initial temperature during distance measurement *($\theta_{i}$* = 25 °C), respectively, with *Δθ = $\theta_{m}$−$\theta_{i}$* and dθ being the uncertainty associated with the temperature values of $\theta_{m}$ and $\theta_{i}$ (dθ = 2 °C). The resolution of the caliper used for distance measurements is 0.01 mm. Its maximum indication error and maximum repeatability error are 0.03 mm and 0.02 mm, respectively.

#### 2.6.3. Uncertainty in Absolute Velocity

Finally, the uncertainty associated with the velocity measurement ($V_{p}=\frac{d}{t_{p}}$) is calculated by applying the following law:

(20) $$u_{c}^{2}\left(V_{p}\right)={\left(\frac{1}{t_{p_{i}}}\right)}^{2}u_{c}^{2}\left(d_{i}\right)+{\left(\frac{d_{i}}{t_{p_{i}}^{2}}\right)}^{2}u_{c}^{2}\left(t_{p_{i}}\right),$$

where $\left(t_{p_{i}}\right)$ and *d_i_* are the corrected values of time and distance measurements, respectively, for one ultrasonic measure in concrete. The corrected values of the ultrasonic velocity measurements are illustrated in the following paragraphs for all concrete samples, with the corresponding error bars.

#### 2.6.4. Uncertainty in Relative Velocity

On the other hand, the uncertainty associated with the velocity variation ($\frac{\Delta V}{V}$) obtained from the correlation results is estimated on the same couple of transducers as follows:

(21) $$\frac{\Delta V}{V}=\left(\frac{V_{i}-V_{\mathrm{ref}}}{V_{\mathrm{ref}}}\right)=\left(\frac{t_{p_{ref}}}{t_{p_{i}}}-1\right),$$

(22) $$u_{c}^{2}\left(\frac{\Delta V}{V}\right)=\left(\frac{t_{p_{i}}^{2}+t_{p_{ref}}^{2}}{t_{p_{i}}^{4}}\right)u_{c}^{2}\left(t_{p_{i}}\right),$$

where $u_{c}^{2}\left(t_{p_{i}}\right)$ is the value of uncertainty associated with the propagation time of the wave ($t_{p_{i}}$) calculated using Equation (13). $V_{ref}$ and $t_{p_{ref}}$ are the velocity and the propagation time of the wave, respectively, within concrete at time *t = t*0. In the next part, we go into further detail about the practical application of these methodologies. Specifically, we use the equations presented above to quantify the uncertainty associated with both the propagation time of the wave and the propagation distance within the concrete structure. By doing so, our goal is to provide a comprehensive evaluation of the reliability of our measurements. Moreover, the uncertainties (*U*) derived from these equations are visually represented alongside data points in graphs in the subsequent section. This not only improves the clarity of our measurements but also facilitates a deeper understanding of the essential uncertainties associated with our measurements. Through this study, we aim to offer valuable insights into the reliability and accuracy of our measurement techniques in real-world applications.

In conclusion, the uncertainty associated with the onset time of the wave is found to be around 1.7%. This comparatively higher uncertainty is due to repeatability, temporal resolution, calibration, and temperature variations. These influence quantities were thoroughly evaluated, and the overall uncertainty reflects the combined effect of these factors. For distance measurements, the uncertainty is determined to be around 0.4%. This inferior uncertainty can be attributed to the precise calibration and resolution capabilities of used measurement instruments and the reliability of the embedded transducer positions within the concrete blocks. Comparing the uncertainties of time and distance measurements highlights the varying levels of precision attainable in the structural diagnosis process. A higher uncertainty in time measurements indicates a need for attentive consideration of temporal factors in the analysis, as a lower uncertainty in distance measurements underlines the reliability of spatial estimation in this study.

These evaluated uncertainties are visually represented, along with data points, in the following sections, enhancing the clarity and comprehension of our results. This visual representation helps to facilitate a deeper understanding of the most significant uncertainties associated with our measurements and ensure that our diagnostics are accurate and reliable.

By acknowledging and quantifying these uncertainties, we can improve the precision of our measurements, advancing the diagnosis of concrete structures using ultrasonic measurements.

## 3. Results

The three blocks were equipped with two embedded transducers (Section 2), generating compression waves with a center frequency of 130 kHz that consist of a sinusoidal with six alternations. The blocks were exposed to temperature variation in a range of 20 to 40 °C, while *V_p_* was measured continuously using the monitoring system presented in the previous section. After setting the temperature to each of the pre-defined values (20, 30, and 40 °C), the temperature of the climate chamber was kept constant for 20 h to ensure that the blocks reached temperature equilibrium throughout.

### 3.1. Influence of Temperature on the Absolute V_p_ Value

**Figure 13:**
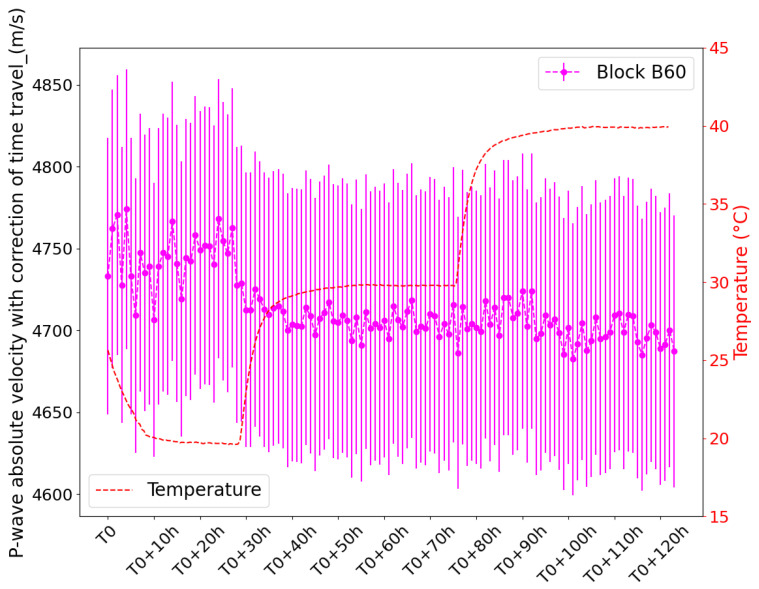
Hourly monitored wave velocity variation (absolute value) with temperature changes in concrete block B60, displaying recorded and corrected values alongside hourly data with associated error bars.

To monitor temperature variation in concrete, signals were recorded on an hourly basis during the temperature variation campaign using embedded piezoelectric transducers. The arrival times of the P wave were determined by applying the AIC technique to the recorded signals, and the distance between the transducers was measured before concrete casting. Figure 13 illustrates how the absolute value of *V_p_* evolves for block B60 when the temperature varies between T0 (laboratory ambient temperature) and 40 °C. As the temperature begins to increase from ambient lab conditions to 40 °C, we observe a notable decrease in the absolute velocity measured during the first hours of monitoring, showing that concrete properties evolve in response to the temperature. However, over the monitoring period, the successive velocity values exhibit fluctuations, with an overall decreasing trend, indicating the influence of measurement noise. The significant level of uncertainty associated with these measurements, as discussed in the previous paragraph regarding absolute velocity uncertainties, makes it challenging to accurately determine the temperature dependency of wave velocity from these data alone. This uncertainty is due to several factors, particularly the resolution of the measurement due to the heterogeneity of concrete, which induces wave scattering and damping. The irregularity of *V_p_* values and high uncertainty highlight the need to improve measurement techniques and use more sophisticated models to obtain a more reliable interpretation of temperature-dependent changes in wave velocity. These results show that the measurement resolution is not sufficient to detect accurate and consistent absolute velocity measurements and that advanced error correction models are necessary to provide reliable data interpretation.

**Figure 14:**
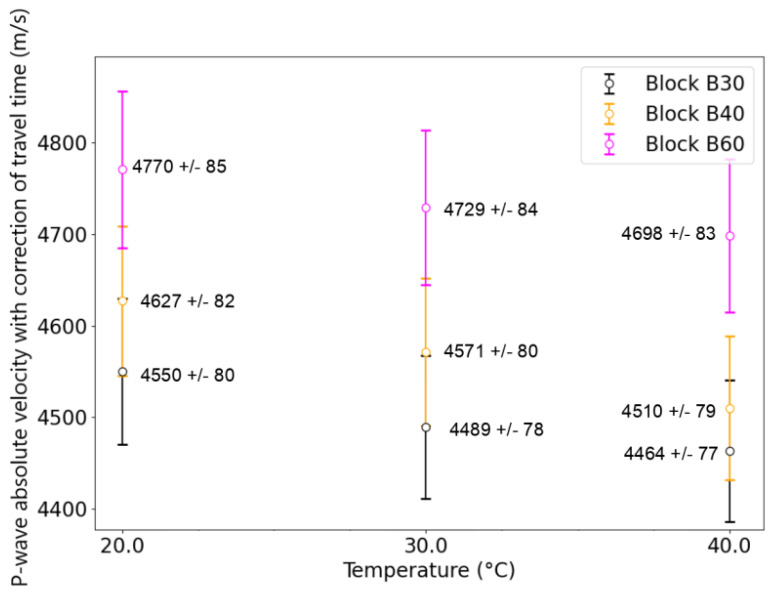
Study of *V_p_* with temperature changes in three different concrete blocks using embedded ultrasonic transducers. Errors bars are computed using Equation (9) (Section 2.6).

Figure 14 presents the average and standard deviation of *V_p_* values at four specific temperatures (20 °C, 30 °C, and 40 °C) for all three blocks, namely B30, B40, and B60. These values were obtained by averaging the values of *V_p_* shown in Figure 13 over a period of approximately 24 h around each temperature step, during which the temperatures were in a steady state. The average *V_p_* generally decreases when the temperature increases from 20 °C to 40 °C, although the measurement uncertainty is about as large as the observed decrease. The average linear rate of decrease is found to be −3.1 [(m/s)/°C], −3.59 [(m/s)/°C], and −5.91 [(m/s)/°C] for blocks B30, B40, and B60, respectively.

The three blocks are different in terms of absolute *V_p_* and temperature dependency. These variations reflect the inherent variability in concrete’s material properties. In sum, our results highlight the level of uncertainty in *V_p_* measures and demonstrate the influence of temperature, as well as the difficulty of obtaining absolute velocity measures during monitoring. Consequently, it is necessary to use relative velocity variation to reduce this issue.

### 3.2. Influence of Temperature on the Relative Wave Velocity Change

**Figure 15:**
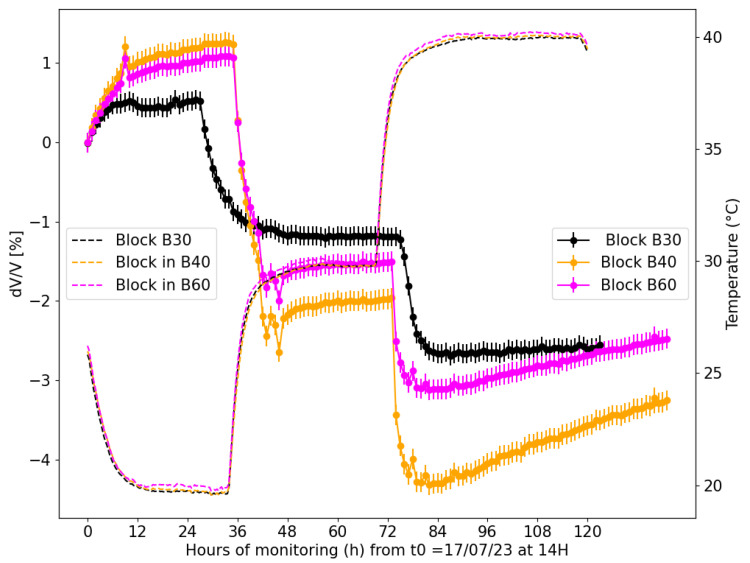
Velocity variation (percentage of reference value) for temperature changes of three samples with different concrete properties (Table 2). The solid lines with error bars represent the variation of velocity (*dV*/*V*), and the dashed lines represent temperature variations in each concrete block.

Figure 15 shows the relative *V_p_* variation with temperature for the three concrete blocks calculated following the procedure described in Section 2.5.2. The change in wave velocity is normalized by *V_p_* at 25 °C when the blocks are first placed in the climate chamber. At the beginning of the experiment, the velocity gradually increases while the blocks’ temperature decreases to 20 °C from the initial 25 °C. The temperature cycle is described in Figure 13. Changing the chamber’s temperature progressively from 20 °C to 30 °C, then to 40 °C, we observe a significant velocity drop of about 1.6 % for block B60, 2.5% for block B40, and 2% for block B30 when the temperature increases by about 30 degrees.

Results show that the influence of temperature variation on *V_p_* measurements is independent of our concrete mixes when the saturation level remains the same (here, 100%). For example, a 20 °C increase in temperature can lead to a decrease in velocity of approximately 2% to 4%, depending on the type of concrete.In comparison, the typical measurement errors associated with ultrasonic techniques, depending on the equipment and conditions, generally range between 0.1% and 0.5%.

This comparison highlights that the variations in *V_p_* due to temperature changes are significantly larger than the measurement errors, underscoring the importance of correcting ultrasonic data for temperature effects in SHM systems. Hence, ultrasonic velocity measurements could be a dependable tool for the SHM of various concrete types when carefully calibrated and corrected for temperature effects. The capacity to detect small variations in ultrasonic *V_p_* is one of the aims of SHM applications, as it can offer early notice of structural degradation, such as pathologies or microcracking, and changes in material properties, leading to structural damage. The observed decrease in *V_p_* with increasing temperature in different concrete mixtures shows the necessity of considering environmental factors like temperature when analyzing ultrasonic data in SHM systems. Therefore, this study suggests that embedded ultrasonic measurement systems can be used for the continuous monitoring of concrete structures by providing real-time data on changes in material properties. However, taking into account measurement uncertainties and implementing temperature correction protocols in the interpretation is essential to effectively use ultrasonic velocity measurements as an SHM metric.

**Figure 16:**
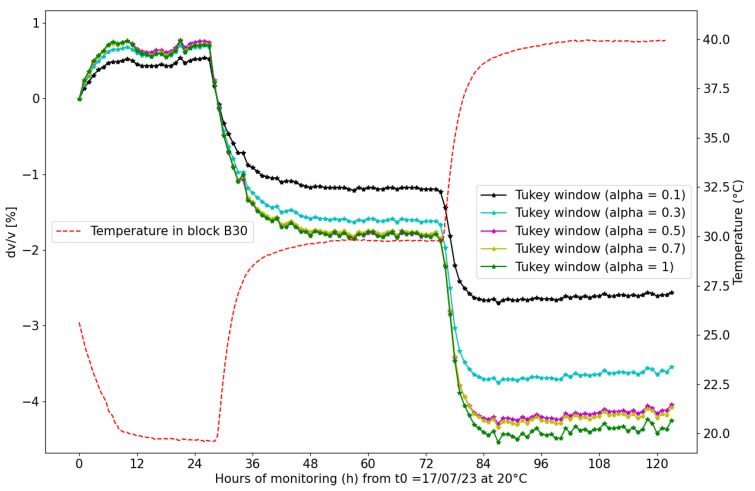
Velocity variation (percentage of reference value) obtained using cross-correlation for temperature changes in concrete block B30. The black line represents temperature variations recorded inside the concrete using an embedded transducer. Tukey windows with different alpha values are applied to the first arrivals of the recorded signals (The size of the Tukey window is kept constant, and the endpoint of the window is always positioned on the 3rd zero (point (1) in Figure 10b)). For each Tukey window (depending on the alpha value), the variation of wave velocity *(dV*/*V*[%]) is presented.

Figure 16 illustrates the influence of the alpha parameter of the Tukey window on the relative change in velocity (*dV*/*V*) with temperature in block B30. The visible differences between the resulting (*dV*/*V*) evolutions demonstrate the sensitivity of the calculated velocity variations to the choice of window, highlighting the importance of careful signal processing in velocity measurements associated with small variations.

## 4. Discussion

Our investigation highlights diverse strategies for the calculation of absolute wave velocities, each accompanied by essential uncertainties requiring careful consideration. Absolute velocity measurements are susceptible to major uncertainties, such as those related to the calibration of transducers, environmental conditions, and fundamental material variability. However, by focusing on relative changes in wave velocity, many of these uncertainties can be minimized, delivering more reliable data for the monitoring of concrete.

By applying Equation (9) (Section 2.6), we can calculate the overall uncertainty (U) associated with the observables (for both absolute and relative velocities), as shown by the error bars in the previous graphs. In each case, the overall uncertainty (U) combines the uncertainties related to time and distance measurements while taking into account all the significant influence quantities, such as temperature, calibration, and repeatability, as explained in Section 2.6.

For example, for each operation of time measurement (through transmission on marble and transducers embedded in concrete), each measurement was repeated 15 times at the same point to calculate a standard deviation for repeatability. The temporal resolution of the signal (dt) was set to 0.001 ms (1 µs), which is related to the resolution of the oscilloscope. Each pair of transducers was calibrated to estimate the error in the wave’s travel time for each transducer. The uncertainty associated with this calibration is included in the overall uncertainty (U).

**Figure 17:**
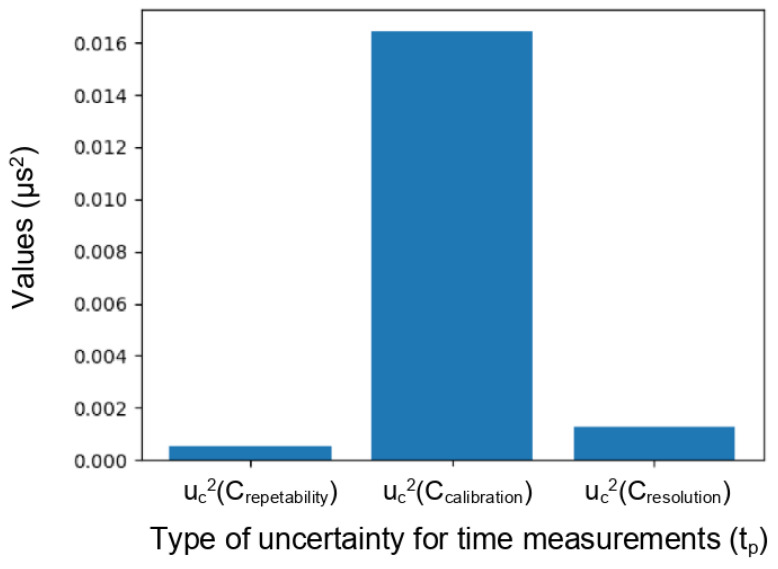
Analysis of factors influencing the measurement of wave propagation time measurement. Transducer calibration uncertainty is identified as the dominant influence.

Figure 17 illustrates the significance of each influential parameter, with the wave propagation time correction associated with the calibration of the transducers being the most significant factor in measurement uncertainty. Calibration uncertainties can significantly influence the interpretation of absolute velocity values. However, for relative velocity, the uncertainty associated with the calibration does not affect the values of *dV/V* because the relative value focuses on the change in velocity rather than the absolute value. Additionally, it is important to acknowledge the difficulty in finding a stable reference material that closely matches concrete in terms of both velocity and attenuation characteristics. This underlines the complexity and significance of transducer calibration in our research with respect to obtaining reliable results.

Unlike the results obtained for the calculation of absolute values, the uncertainty associated with the values of *dV/V* shows minimal variance concerning the velocity evolution due to temperature (Figure 15).

The use of relative velocity change allows for continuous monitoring and comparison with higher accuracy over time. The decrease in measurement uncertainty, from the important levels found in absolute velocity measurements to around 0.12% in relative measurements, underlines the accuracy of this approach. Furthermore, the data show that the relative velocity change is persistent across different concrete mixes, indicating general applicability for the monitoring of various concrete structures. In summary, the transition to relative velocity variation significantly improves the accuracy and applicability of ultrasonic monitoring in concrete structures. This technique, assisted by the reduced uncertainty and consistent data across different concrete mixes, demonstrates the potential for more effective and reliable monitoring using embedded ultrasonic transducers.

Additionally, we find that the size of the Tukey window used for cross-correlation significantly influences the results. Changing the size of the window results in variations in the calculated relative velocities. This is because the information extracted within the applied window influences the correlation process. When a smaller window is used, primarily focusing on the arrival of the signal, results found in time variation are based essentially on the initial wavefront. However, considering a larger window extending beyond the first arrival of the signal may provide additional relevant information related to additional waveforms, such as S waves or other subsequent arrivals. Finally, the deployment of our developed ultrasonic embedded measuring system demonstrates its efficacy in real-time concrete monitoring, particularly under fluctuating temperature conditions.

## 5. Conclusions

This study presents the temperature dependency of ultrasonic wave velocity in concrete measured using embedded transducers while taking into account measurement uncertainties.

In this study, we developed ultrasonic transducers for embedding in concrete, overcoming challenges like interference noise. Shielding and protective layering helpfully reduced capacitive coupling interferences derived from the high water content of concrete. Calibration procedures using a marble sample ensured accurate measurement of the P-wave propagation time, underlining the precision of our embedded ultrasonic measurement system. The developed transducers were embedded in three different concrete mixes, namely B30, B40, and B60. This experiment helped to evaluate ultrasonic wave velocity under temperature variations ranging from 20 °C to 40 °C. The evaluation of uncertainties associated with the ultrasonic measurements and the visualization of these uncertainties alongside our data provide a comprehensive assessment of measurement reliability.

This research underlines the sensitivity of both absolute wave velocity and relative wave velocity change to small temperature variations in concrete. Results show that although it is challenging to measure absolute wave velocity with sufficient precision to trace minor variations due to the high uncertainty, relative wave velocity measurements are essential and more dependable for the monitoring such small changes. According to our findings, a 10 °C increase in temperature decreases the velocity by an average of 30 m/s.

The uncertainties associated with both absolute and relative velocity measurements were thoroughly analyzed, showing minimal variance in relative velocity measurements and highlighting the reliability of relative velocity change as a monitoring technique. Finally, our experimental setup and methodology showed the ability to address these challenges and proved effective in reliably detecting small velocity changes in concrete.

The validation of the ultrasonic measurement system with embedded transducers proposed in this study provides the foundation for the development of a multi-sensor measurement system to monitor the most important indicators of concrete and their gradients. Thus, following this work, we will focus on developing an ultrasonic measurement system comprising several pairs of piezoelectric transducers embedded at different depths within the concrete to monitor the evolution of durability indicators and their gradients, specifically the water content gradient within the concrete. Our ultimate goal is to invert the observables, such as the wave velocity profiles, with the evaluation of indicators associated with concrete structure, such as Young’s modulus, porosity, and water content.

Moreover, this study paves the way for new avenues in the SHM of concrete structures using embedded ultrasonic technologies. Future investigations will prioritize improving the quality of ultrasonic measurements and reducing measurement uncertainties, facilitating real-time monitoring of concrete structures under diverse environmental conditions. By doing so, we aim to extend the exploitation period of concrete structures by computing a detailed diagnosis and a recalculation of the service life duration of these structures.

## Figures and Tables

**Table 1:** Comparison between PZT, PVDF, quartz, and AlN piezoelectric sensors used in SHM [36].

Piezoelectric Transducers	PZT (Lead Zirconate Titanate)	PVDF (Polyvinylidene Fluoride)	Quartz (SiO_2_)	AlN (Aluminum Nitride)
**Material**	Lead zirconate titanate (PZT)	Polyvinylidene fluoride (PVDF)	Quartz (silicon dioxide)	Aluminum nitride
**Design**	Typically bare or with minimal protective coatings	Flexible polymer film	Rigid ceramic or crystal	Rigid ceramic
**Interference Mitigation**	Basic shielding, more susceptible to capacitive noise	Susceptible to electromagnetic interference	High resistance to interference	Good resistance to interference, can be sensitive to temperature
**Calibration Requirement**	Standard calibration, generally less complex	Requires careful calibration due to flexibility	Generally stable but needs calibration for precision	Calibration needed for specific applications, temperature-sensitive
**Frequency Range**	kHz to several MHz, depending on the design	1 kHz to 100 MHz, typically used in lower-frequency applications	1 kHz to several MHz, depending on the crystal cut	100 kHz to several MHz, depending on the crystal cut
**Sensitivity**	High sensitivity, excellent for precise measurements	Lower sensitivity but flexible	High sensitivity, stable performance	High sensitivity and stable under varying conditions
**Environmental Suitability**	Robust but contains lead and may need additional protection for harsh environments	Flexible and suitable for dynamic surfaces but less durable	Very stable and suitable for harsh environments	Stable and durable but can be sensitive to temperature changes
**Applications in SHM**	Crack detection, delamination monitoring, and impact detection	Distributed strain sensing and vibration monitoring on flexible structures	Precision measurements and high-frequency applications	High-temperature environments and precision measurements

**Table 2:** Description of concrete blocks and their properties.

Description of the Blocks	B30	B40	B60
Geometry of the blocks (L × l × h) (cm)	30 × 30 × 30	30 × 30 × 30	30 × 30 × 30
Distance between transducers (cm)	10.01	9.98	10.03
Density (kg/m^3^)	2401	2408	2436
Young’s Modulus E (MPa)	35,719	37,321	43,279
Compressive strength (MPa)	34.4	46.9	64.2

## Data Availability

The data presented in this study are available on request from the corresponding author due to privacy reasons.
